# The Conundrum of Low COVID-19 Mortality Burden in sub-Saharan Africa: Myth or Reality?

**DOI:** 10.9745/GHSP-D-21-00172

**Published:** 2021-09-30

**Authors:** Janica Adams, Mary J. MacKenzie, Adeladza Kofi Amegah, Alex Ezeh, Muktar A. Gadanya, Akinyinka Omigbodun, Ahmed M. Sarki, Paul Thistle, Abdhalah K. Ziraba, Saverio Stranges, Michael Silverman

**Affiliations:** a Department of Epidemiology and Biostatistics, Schulich School of Medicine and Dentistry, Western University, London, Ontario, Canada.; b Department of Medicine, Schulich School of Medicine and Dentistry, Western University, London, Ontario, Canada.; c Public Health Research Group, Department of Biomedical Sciences, University of Cape Coast, Cape Coast, Ghana.; d Dornsife School of Public Health, Drexel University, Philadelphia, PA, USA.; e School of Public Health, University of the Witwatersrand, Johannesburg, South Africa.; f Bayero University, Kano, Kano State, Nigeria.; g Aminu Kano Teaching Hospital, Kano, Kano State, Nigeria.; h University of Ibadan, Ibadan, Nigeria.; i College of Medicine, University of Ibadan, Ibadan, Nigeria.; j Pan African University Life & Earth Sciences Institute (PAULESI), Ibadan, Nigeria.; k School of Nursing and Midwifery, Aga Khan University, Kampala, Uganda.; l Family and Youth Health Initiative (FAYOHI), Jigawa State, Nigeria.; m Karanda Hospital, Mount Darwin, Zimbabwe.; n The University of Zimbabwe, Harare, Zimbabwe.; o University of Toronto, Toronto, Canada.; p African Population and Health Research Center, Nairobi, Kenya.; q Department of Family Medicine, Western University, London, Ontario, Canada.; r The Africa Institute, Western University, London, Ontario, Canada.; s Department of Population Health, Luxembourg Institute of Health, Strassen, Luxembourg.; t Division of Infectious Diseases, Western University, London, Ontario, Canada.

## Abstract

The demographic age structure of sub-Saharan Africa contributes significantly to the low morbidity and mortality of COVID-19 compared to other regions in the world.

## Key Messages

- Evidence suggests the demographic age structure of sub-Saharan Africa is the leading factor of the low morbidity and mortality of COVID-19 compared to other regions of the world.
- Widespread social mitigation strategies, such as lockdowns, have resulted in severe economic and societal consequences in terms of food security, adolescent pregnancy, gender-based violence, and disruptions in treating other diseases.
- It is imperative to weigh the risks and benefits of social mitigation strategies for future waves.

## BACKGROUND

COVID-19 has impacted the world immensely since its discovery in the city of Wuhan, China, in December 2019.^1^^,^^2^ As of June 27, 2021, approximately 181.9 million COVID-19 cases have been confirmed with more than 3.9 million deaths.^3^ COVID-19 has dramatically impacted the Americas, Europe, and Asia. As of June 27, 2021, in the Americas, 73.1 million confirmed COVID-19 cases with 1.9 million deaths have been reported, 47.8 million confirmed cases with more than 1 million deaths in Europe, and 55.4 million confirmed cases with 784,965 deaths in Asia.^4^

The impact of COVID-19 in Africa has been substantially lower compared to countries in the Americas, Europe, and Asia. The World Health Organization (WHO) African Region reported more than 3.9 million confirmed cases and 94,217 deaths, as of June 27, 2021.^5^ Moreover, the mortality rate of COVID-19 per million in Africa is considerably lower than in all other WHO regions other than the Western Pacific (Table 1).^5^^–^^11^ Public health preparedness is a significant aspect in the success of reducing COVID-19 transmission. Lessons learned from countries across Eastern Asia imply the need for community-oriented strategies and rapid response from public health officials to successfully contain the COVID-19 pandemic.^12^ Strategies such as early case identification, widespread laboratory testing and screening, outbreak mitigation (up to and including lockdowns), contact tracing, health education, physical distancing, and quarantine measures have been demonstrated as essential interventions in curbing the pandemic.

**TABLE.** Confirmed COVID-19 Cases and Mortality Rates per WHO Region^a^

**WHO Region** ^b^	**COVID-19 Cases**	**COVID-Related Deaths**	**Population** ^c^	**Mortality Rate per Million**
Africa^5^	3,942,448	94,217	1,019,922,000	92.4
Americas^6^	71,959,063	1,891,291	992,155,000	1,906.2
South-east Asia^7^	34,657,785	485,398	1,947,632,000	249.2
Europe^8^	55,821,905	1,181,992	916,315,000	1,289.9
Eastern Mediterranean^9^	10,916,353	215,799	664,336,000	324.8
Western Pacific^10^	3,521,244	54,069	1,889,901,000	28.6

Abbreviations: COVID, coronavirus disease; WHO, World Health Organization.

a Information up to date as of June 27, 2021.

b Refer to the Supplement for a comprehensive list of WHO Member States.

c Population data taken from the 2016 WHO Global Health Observatory data repository.^11^

Lessons learned from Eastern Asia imply the need for community-oriented strategies and rapid response from public health officials to successfully contain the COVID-19 pandemic.

This article critically examines the hypotheses that have been attributed to the apparently lower than expected morbidity and mortality of COVID-19 in SSA to help guide public health decision making regardin g essential interventions for containing COVID-19.

## POTENTIAL MITIGATING FACTORS INFLUENCING THE MORBIDITY AND MORTALITY OF COVID-19 IN SUB-SAHARAN AFRICA

**FIGURE 1:**
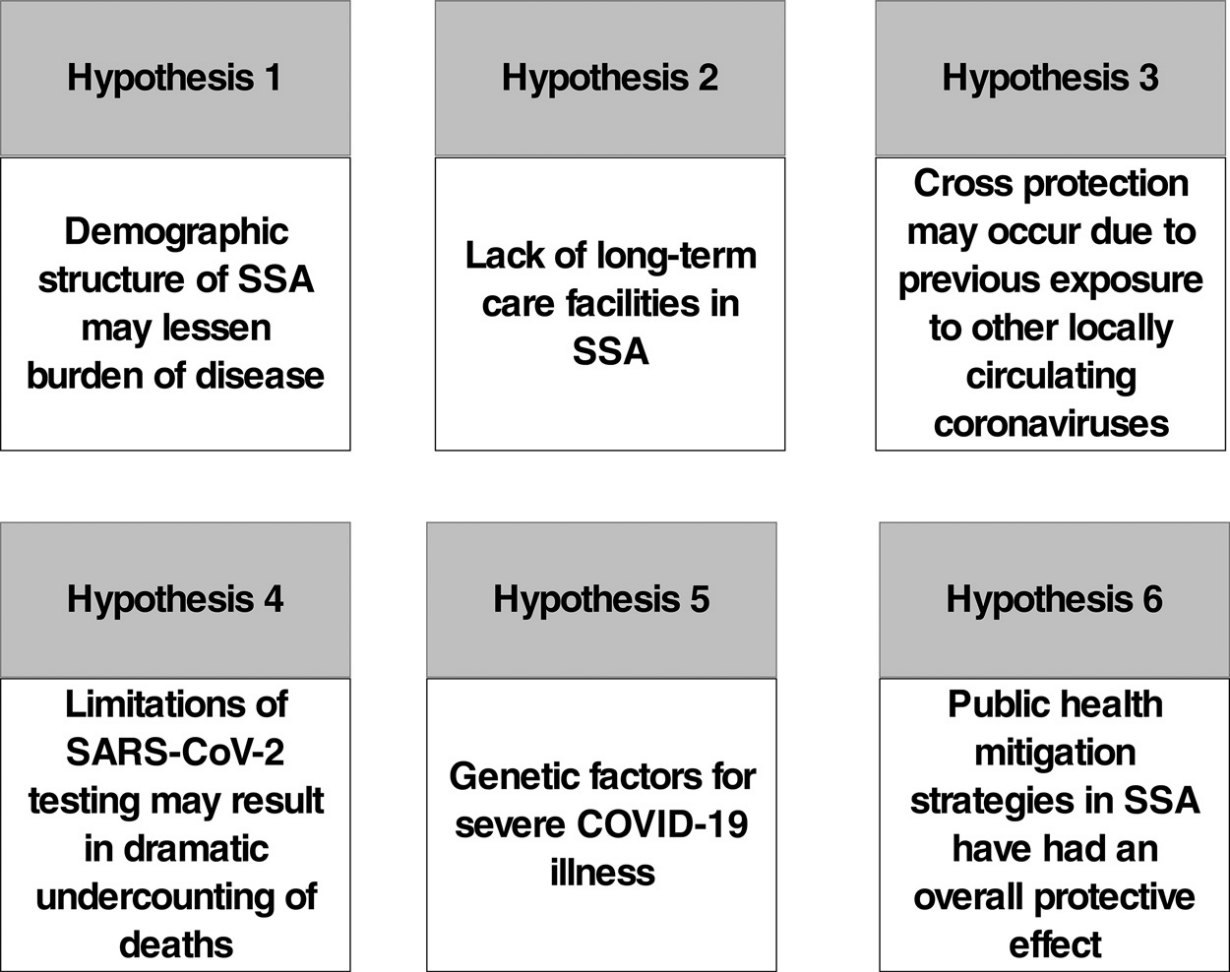
Proposed Hypotheses Explaining the Limited Impact of COVID-19 in SSA Abbreviations: COVID-19, coronavirus disease; SARS-CoV-2, severe acute respiratory syndrome coronavirus 2; SSA, sub-Saharan Africa.

It is posited that the low impact of COVID-19 in SSA is due to 1 or several of 6 main hypotheses (Figure 1).

### Hypothesis 1: Demographics of sub-Saharan Africa

Global mortality trends of COVID-19 show marked differences by demographic characteristics including age (increased risk of severe illness in older individuals), sex (higher among males), socioeconomic status, and race (higher among Blacks). In the United States, the Centers for Disease Control and Prevention (CDC) report that 80% of COVID-19-related deaths occur in individuals aged 65 years and older.^13^^,^^14^ Data from the United Kingdom has demonstrated that the strongest risk for death is advanced age, which dramatically outweighs the risks associated with any other demographic factor or medical condition.^15^ Demographic structures for Europe, the Americas, and Asia demonstrate median age ranges from 32 years to 42.5 years,^4^^,^^16^^–^^19^ with 8.9% to 19.1% of the population older than 65 years.^20^^–^^23^

**FIGURE 2:**
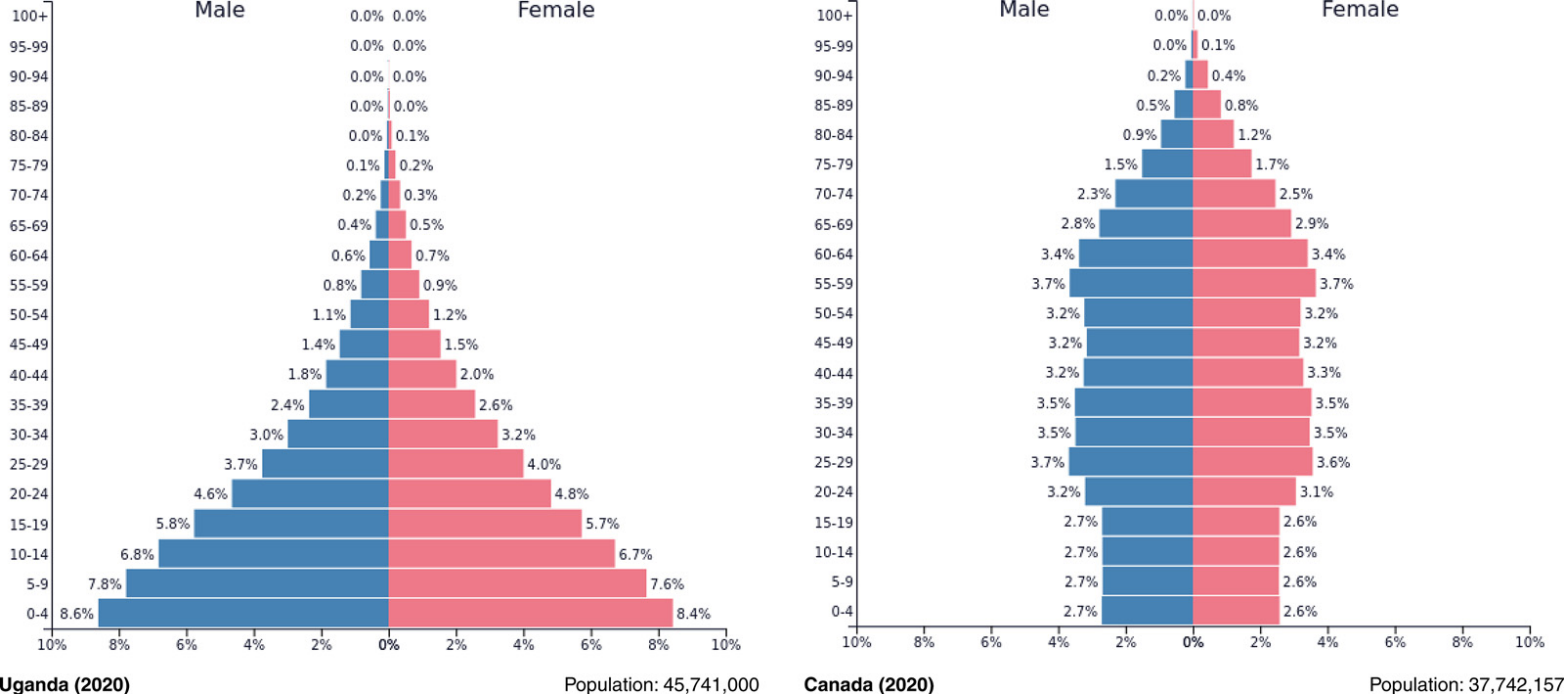
Population Pyramids of Uganda and Canada^28^^,^^29^

**FIGURE 3:**
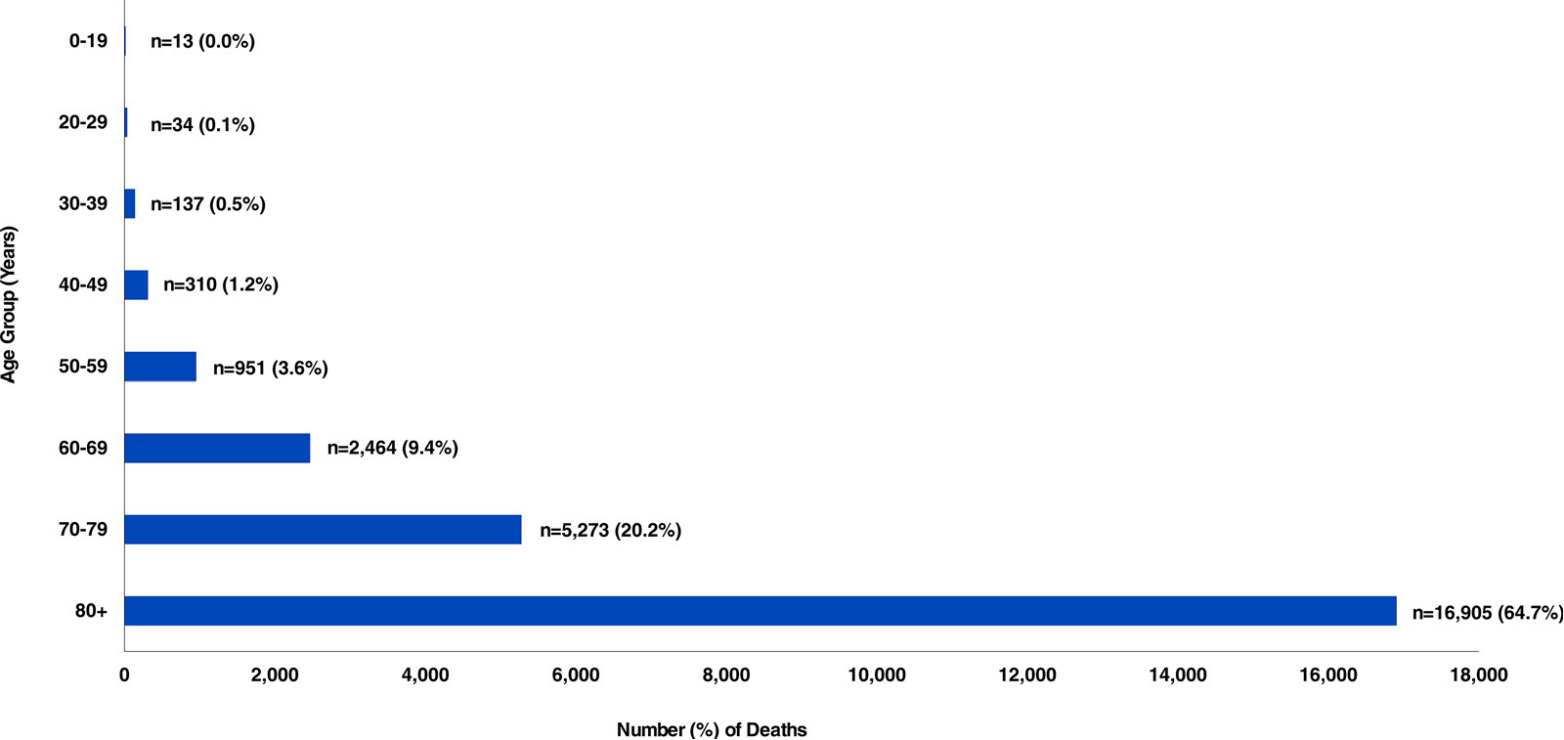
Age Distribution of COVID-19 Cases Deceased in Canada as of June 25, 2021^30^

In contrast, the median age of the SSA population is considerably lower, with a median age of 18 and only 3.0% of the African population older than 65 years.^24^^,^^25^ Figure 2 compares the population pyramids of Uganda and Canada, which are similar in overall population size. The median age of Canada (41.1 years) is remarkedly higher than that of Uganda (16.7 years).^26^^,^^27^ In Uganda, less than 0.2% of the population is in the highest-risk group of developing more severe illness (aged 80 years and older).^28^ Conversely, the proportion of individuals aged 80 years and older in Canada is higher (4.4%).^29^ Further, Figure 3 illustrates the distribution of COVID-related deaths in Canada as of June 25, 2021.^30^ A large proportion of deaths are attributed to older age; approximately 98.0% of COVID-related deaths occur in individuals aged 50 years and older, with approximately 64.7% in individuals aged 80 years and older.^30^ With the rollout of COVID-19 vaccinations and prioritization of those aged 70 years and older in North America and other areas, the mean age of those being admitted to hospital has decreased.^31^^,^^32^ However, it is still highly likely that those aged 70 years and older remain the highest risk among the unvaccinated population.

Comparison of the age demographics of Uganda with other lower-middle-income countries in regions such as Latin America and the Caribbean and South Asia demonstrates the uniqueness of the demographic structure in SSA. The median age in Brazil is 33.5 years, Peru 31.5 years, and Mexico 29.2 years, which are all markedly higher than in SSA. Low-income countries in Latin America and the Caribbean, such as Nicaragua, El Salvador, and Haiti also have greater median ages (24.0–27.6 years) and a larger proportion of the population age 65 and older (5.2%–8.7%) than in SSA.^33^^–^^38^ Similar demographics are observed for countries in South Asia, such as India and Pakistan; median age ranges from 22.8–28.4 years with 4.4%–6.6% of the population aged older than 65 years.^39^^–^^42^

Older age is associated with more degenerative and metabolic disorders that have also been shown to heighten the risk of death from COVID-19. Therefore, it is posited that the demographic structure of SSA plays a critical role in the low morbidity and mortality of COVID-19. It is possible that the burden of severe disease and death may be low despite suspected and undetected widespread transmission. In fact, it is possible that widespread transmission has already occurred without precipitating the high death rates seen elsewhere due to the relatively small proportion of elderly and lack of large long-term care facilities for the elderly, which have been the epicenters of mortality in Canada and elsewhere.^43^ It is notable that some areas of SSA, such as South Africa, have a much higher median age (27.6 years), which could be a reason for the higher COVID-19 death rates seen there.^44^

It is possible that widespread transmission has already occurred without precipitating the high death rates seen elsewhere due to the relatively small proportion of elderly and lack of large long-term care facilities.

### Hypothesis 2: Lack of Long-Term Care Facilities

In addition to the demographic pyramid demonstrating very low numbers of elderly, the elderly in SSA do not tend to live in long-term care facilities. The CDC defines long-term care facilities as those whereby elderly who are unable to live independently receive medical and personal care.^45^ Unfortunately, long-term care facilities pose a significant risk for infectious and communicable diseases; approximately 1.0–3.0 million infections occur in these facilities per year.^45^^,^^46^ During the first wave of the epidemic in Canada, 81.0% of all deaths occurred in long-term care facilities.^47^ Transmission to the elderly can be particularly efficient in these settings and lead to a markedly higher infection fatality rate.^48^

Across SSA, long-term care facilities are almost nonexistent, with the notable exception of South Africa, leaving the provision of care to families.^49^^,^^50^ Large young families with high levels of unemployment and low labor costs enable care to be provided by individual relatives rather than a team of professionals, which limits the number of caregivers that may transmit infection. In the first wave, approximately 33% of South African long-term care facilities experienced outbreaks.^51^ Furthermore, data from South Africa have demonstrated that COVID-19-related deaths are highly correlated with increased age; approximately 2.2% of all COVID-19-related deaths occurred among persons younger than 30 years, despite their consisting of 54.2% of the population.^52^^,^^53^ This is a further potential explanation for South Africa being an outlier with a higher death rate than in other African countries.^54^

### Hypothesis 3: Prior Exposure to Coronavirus Infection

In addition to severe acute respiratory syndrome coronavirus 2 (SARS-CoV-2), the virus that causes COVID-19, 6 other human coronaviruses have been identified. Seasonal human coronaviruses, such as NL6, 229E, OC43, and HKU1, are common and result in cold- or flu-like symptoms.^55^ Zoonotic coronaviruses, such as Middle East Respiratory Syndrome (MERS)-CoV and SARS-CoV, are responsible for more severe diseases.^55^ Previous exposure to locally circulating coronaviruses and the development of antibodies is posited to mediate cross-protection to COVID-19 and induce partial immunity.^56^

Several studies have been conducted to investigate this unique relationship. Studies assessing antibody prevalence to SARS-CoV-2 in pre-pandemic serum samples observed a significant increase in the prevalence of cross-reactivity among sera in SSA compared to other continents.^57^ In addition, previous studies have demonstrated high false positivity when testing pre-pandemic sera from SSA using European assays.^58^^,^^59^ The discrepancy of seropositivity may be attributed to widespread exposure to various endemic coronaviruses before the emergence of the SARS-CoV-2 pandemic. A limitation of these studies was the use of serological assays to determine previous exposure, particularly because there can be discrepancies in results when comparing T-cell versus antibody evidence of exposure and immunity.^60^ In contrast, a study by Sagar et al.^61^ used results from previously performed comprehensive respiratory panel polymerase chain reaction assays to examine the impact of previous exposure to endemic coronaviruses in COVID-19 patients. Their results demonstrated a significant decrease in odds of mortality and odds of being admitted to an intensive care unit in patients who had evidence for previous exposure to endemic coronaviruses compared to those who did not.^56^^,^^61^ These findings indicate that exposure to other coronaviruses may reduce the severity and burden of COVID-19. Furthermore, a recent study by Uyoga et al.^62^ observed increased rates of antibody prevalence to SARS-CoV-2 among Kenyan blood donors between April 30–June 16, 2020, that are higher than case counts would predict.

### Hypothesis 4: Limited Access to Adequate Testing

There are concerns regarding the recording of COVID-19 cases in SSA. It is hypothesized that there has been a dramatic undercounting of deaths due to lack of SARS-CoV-2 testing as was suggested in the mass media to have happened in Kano, Nigeria.^63^^,^^64^ Current data may not reflect the true extent of the disease. The true numbers of infected and deaths could be higher given that, at least in South Africa where the median age is much higher than SSA as a whole,^44^ the excess mortality observed is far higher than the officially reported totals for deaths from COVID-19. Lack of local access to testing and contact tracing, and insufficient data collection have interfered with the ascertainment of the incidence and prevalence of COVID-19 in SSA. The WHO reports varying levels of testing across Africa, however, testing is still relatively low compared to other areas of the world.^65^ As of June 25, 2021, testing rates ranged from as low as 7.7 tests per 1,000 population in Madagascar to as high as 215.3 and 389.9 tests per 1,000 in South Africa and Gabon, respectively.^66^ However, these numbers are far lower than rates in the United States (1,401.8 tests per 1,000 population) and the United Kingdom (2,973.0 tests per 1,000 population).^66^ Although low testing rates likely resulted in a much lower case rate, the lack of hospital overcrowding and widespread deaths likely resulted from lower morbidity and mortality in this region. This would suggest a lower predisposition to severe illness. The initial priority for the Africa Task Force for Novel Coronavirus was to expand COVID-19 testing capability. This expansion proceeded rapidly; at the outset of the pandemic, only 2 labs in Africa were capable of SARS-CoV-2 detection, but by mid-March 2020, 43 countries had this laboratory capability.^67^ Preliminary observations from the poorly maintained civil and vital registration systems seem to indicate that it is unlikely that there has been excess all-cause mortality in the region.^68^ Studies are underway in Kenya assessing excess mortality through verbal autopsies and population-based serosurveys for past infections to assess past exposure.^69^

There is a hypothesis that deaths have been undercounted due to lack of SARS-CoV-2 testing.

The concerns of recording the impact of COVID-19 across SSA offers the opportunity of novel means of data collection to expand current knowledge on COVID-19 morbidity and mortality. Morbidity may be further explored through the use and purchase of oxygen as a proxy of the current situation in hospitals. Further, data collection on death may be extended to churches and faith groups, obituaries, and morticians. These and other means should be further explored to help better understand the impact of COVID-19 on SSA as a whole.

### Hypothesis 5: Genetic Risk Factors

Studies from developed countries have demonstrated a higher risk of death in racialized communities, including those of African or South Asian descent.^15^ This predisposition is likely related to socioeconomic factors including poverty, crowding, and working in essential services. Therefore, overall environmental exposures are likely far more important than genetic exposures in disease susceptibility.

### Hypothesis 6: Effective Government Public Health Response to COVID-19 Threat

Another hypothesis is that African governments and public health organizations moved remarkably swiftly in response to the threat of COVID-19. Early in January 2020, African governments began to plan for the arrival of COVID-19 as high flight volumes between China and Africa predicted early spread to multiple locations including South Africa, Nigeria, and Kenya.^70^ As early as January 2, 2020, Côte d’Ivoire implemented enhanced screening measures for passengers arriving from China.^71^ Other African countries swiftly followed suit. In February 2020, the first meeting of the newly established Africa Task Force for Novel Coronavirus convened. The first confirmed case of COVID-19 on the African continent was reported in Egypt on February 14, 2020, and linked to travel from China. By March, almost all African nations had suspended flights from China. After March 2020, most cases imported to Africa originated from Europe, as the epicenter of the disease had shifted there.^72^ By May 2020, more than 40 African nations had closed their borders to all but cargo.^71^

Another hypothesis is that African governments and public health organizations moved remarkably swiftly in response to the threat of COVID-19.

National public health institutions are responsible for disease surveillance, diagnostics, and rapid response to outbreaks, making them essential for curbing the emergence and re-emergence of infectious diseases in the African context, especially COVID-19.^73^ As of 2019, Botswana, Ethiopia, Ghana, Liberia, Morocco, Mozambique, Namibia, Nigeria, Rwanda, Sierra Leone, South Africa, Uganda, and Zambia all had highly functional national public health institutions with experience in battling infectious diseases.^73^^,^^74^ These organizations focus on infectious disease threats, which is in contrast to those organizations in high-income countries that have focused on noncommunicable diseases. Uganda is a leading example of curbing the impact of COVID-19 in the African context. Rapid response and implementation of risk communication, testing, social and physical distancing measures, and contract tracing were critical for the success seen in Uganda.^75^

Additionally, new programs to promote regional sharing of COVID-related information were initiated across SSA. For example, the East African Community created the Regional Electronic Cargo and Drivers Tracking System. This system electronically shares the COVID test results of truck drivers between member countries. In addition, the program uses the drivers’ cell phones to track their routes and record stops. This tracking allows for quick contact tracing in the event of a COVID-19 outbreak.^76^ Furthermore, several African countries have scored particularly well in critical policy areas in terms of public health directives, financial responses, and fact-based public communications to help control COVID-19.^77^ In particular, Kenya, Ghana, and Ethiopia scored more than 95 on a 100-point scale.^77^ This may have helped to mitigate the scope of the pandemic although further validation of the scores would be helpful.

### Other Hypotheses

#### Adherence to Preventative Strategies

Some studies suggest that adherence to recommendations for handwashing, social distancing, and public masking has been widespread in SSA,^78^^,^^79^ however, the generalizability of these observations to multiple SSA countries and contexts, as well as comparative data between African and non-African countries require further study.

#### Drugs Against Parasitic Infections

Infections with parasites have been suggested to be associated with less severe COVID-19 in an as yet non-peer-reviewed Ethiopian study although this finding requires replication in other locales.^80^ SSA countries within the tropical and equatorial regions appear to have the lowest proportion of confirmed COVID-19 cases and the highest burden of malaria infection.^81^ Several factors have been posited to contribute to the low incidence of COVID-19 in these malaria-endemic countries, including cross-protection from consistent use of antimalarial medication.^81^ However, the failure of hydroxychloroquine to prevent COVID-19 in randomized studies makes this hypothesis less likely.^82^ In addition, ivermectin, an antiparasitic drug used to treat several neglected tropical diseases, such as onchocerciasis, strongyloidiasis, and lymphatic filaria,^83^^,^^84^ has been widely used across SSA since the 1990s.^85^ A study conducted by Caly et al.^86^ found ivermectin to be an inhibitor of the SARS-CoV-2 virus *in vitro*. Despite the hypothesized association between antiparasitic medications and COVID-19, at present, there is still only limited evidence to support it.^87^^,^^88^

#### Prevalence of Noncommunicable Diseases

Noncommunicable diseases, such as hypertension, diabetes, and obesity, have been observed to increase the severity of COVID-19 illness.^89^ In comparison to North America, the rates of noncommunicable diseases, such as diabetes and obesity, are remarkedly lower in SSA. Data from 2017 demonstrate that the prevalence of diabetes in the United States and Canada was observed to be 10.8% and 7.4%, respectively.^90^ Conversely, the prevalence of diabetes among SSA was observed to range from 1.0%–7.8%, with exception of Sudan and South Sudan, whereby the prevalence of diabetes was 15.7% and 10.4%, respectively.^90^ Further, approximately 70.2% and 67.5% of adults in the United States and Canada, respectively, have been observed to be either overweight or obese (BMI greater than 25).^91^ Conversely, among countries in SSA, these rates range from 18.1%–38.4%, with exception of South Africa whereby 51.9% of adults are either overweight or obese.^91^ The prevalence of hypertension, however, is considerably higher in SSA compared to North America.^92^ Further studies should be conducted to understand the roles of noncommunicable diseases and COVID-19 severity in the African context.

Diabetes and obesity have been observed to increase the severity of COVID-19 illness, but the rates of diabetes and obesity are remarkedly lower in SSA compared to North America.

#### Mobility

It also has been hypothesized that lower mobility and spending a greater amount of time outdoors may have reduced the risk of COVID-19, especially in impoverished rural areas.^93^ Reduced travel between African countries due to limited visa-free relationships may have also limited spread across the continent.^94^ Further study would be necessary to confirm these hypotheses.

## SOUTH AFRICA AS AN OUTLIER

South Africa appears to have had a particularly high incidence of COVID-19 hospitalizations and deaths. This has been attributed to several phenomena. As noted above, South Africa has a higher median age as well as an established long-term care facility sector. The very high HIV and TB burden in South Africa may be another factor as both of these were found to be associated with an increased COVID-19 mortality rate in a South African cohort.^54^ Maintaining antiretroviral therapy is particularly important in light of the data demonstrating poor COVID-19 outcomes in patients with low CD4 counts.^95^ In addition, the effects of noncommunicable diseases may contribute to the higher burden of COVID-19 seen in South Africa. The prevalence of hypertension in South Africa has been reported to range from 26.9%–30.4% and is increasing.^96^^,^^97^ Furthermore, the prevalence of diabetes in South Africa has been reported to be 12.8%^98^ and was found to be the second leading cause of death in South Africa in 2015.^99^ Moreover, obesity rates among men and women in South Africa have been reported to be 31.0% and 68.0%, respectively. Further research needs to be conducted on various noncommunicable factors that may contribute to the increased COVID-19 burden seen in South Africa.

Better diagnostics and health care documentation, including death registries, may also allow for higher reporting rates. The emergence of the SARS-CoV-2 variant, 501.V2, has demonstrated the potential for greater transmissibility and risk of reinfection as well as a concern of relative vaccine resistance, leading to severe future waves of infection in South Africa.^100^^,^^101^

## IMPLICATIONS FOR POLICIES AND PROGRAMS

Based on the current COVID-19 situation in SSA, to help policy makers and programs improve health practice, the following policy prescriptions have emerged:

- Reduce emphasis on lockdowns, which may disproportionately affect young people and the poor and may lead to other severe health consequences as noted in the article.
- Emphasize the importance of good governance regarding health directives and open communication.
- Provide financial support to vulnerable sectors as per experience in Kenya and Ghana.^77^ In light of the limited resources of many African countries, this may require the assistance of external agencies.
- Prioritize an international effort to develop vaccines tailored to the SARS-CoV-2, 501.V2.
- The impact of oxygen shortages in a developing country suffering a COVID-19 outbreak has been severely apparent in India.^102^^,^^103^ Therefore, governments must ensure the availability of medical infrastructure should an unexpected rapid system-wide severe outbreak occur.
- Prioritize efforts to establish molecular epidemiology to be aware of the emergence of new variants. In particular, the emergence of new variants of concern, which may be more virulent in younger populations, would require a reconsideration of Africa’s susceptibility to a severe epidemic.
- Conduct studies to determine the risk factors for severe disease in the African context. These may include detailed cohort studies of patients who do get severely ill in SSA countries with appropriate controls (such as patients who test negative for SARS-CoV-2).

## CONCLUSIONS

In reviewing the totality of the evidence, we believe that it is suggested that in SSA the overall death rate is lower than in most other regions primarily due to the demographic structure with a low median age and a small percentage of vulnerable elderly, although as noted, other factors likely also play a role. Some localized areas with a greater number of older individuals, such as South Africa, may be exceptions to this trend. The presence of a long-term care facility sector as well as extremely high rates of HIV and TB coinfection, and effects of noncommunicable diseases may also have led to South Africa having a higher disease burden. Limited resources for disease diagnosis, effective public health campaigns, and other factors discussed are also important considerations. Further studies to clarify these various hypotheses for the low mortality presently reported in Africa are required. While data accrue, the risks and benefits of widespread social mitigation strategies such as lockdowns, need careful consideration. The continent is reeling from the effects of the pandemic; the economic and societal tolls in terms of hunger, teen pregnancy, gender-based violence, and disruptions in the treatment of malaria, TB, and HIV are enormous. Furthermore, the 501.V2 variant of SARS-CoV-2 heightens the risks of further waves and raises the risk to the rest of the continent, including the danger of hospitals reaching capacity in other SSA countries.^104^ However, as discussed, widespread adoption of stringent lockdown strategies used previously should be undertaken only with great caution. Consideration must be given to local, unique conditions such as the age structure of the population, competing health risks, and food security.^104^

With the recent experience of a severe second wave in India, it is imperative to establish adequate molecular epidemiology to monitor emerging variants that have the potential to cause severe infection in the younger population. As full vaccine rollout in Africa with widespread coverage will likely not occur for some time, these issues remain of critical importance. This review of the literature will aid countries in adopting unique strategies for limiting the spread of COVID-19 without the need for stringent lockdowns. Further research on the potential mechanisms needs to be carried out to understand other possible reasons for the observed discrepancy in mortality seen in SSA.

## Supplementary Material

21-00172-Silverman-Supplement.pdf

## Notes

**Peer Reviewed**

**First published online:** July 15, 2021.
