# Supplementary material for: The Conundrum of Low COVID-19 Mortality Burden in sub-Saharan Africa: Myth or Reality?
Source: Glob Health Sci Pract. 2021 Sep 30;9(3):433–43. doi: 10.9745/GHSP-D-21-00172 (PMC8514030; doi:10.9745/GHSP-D-21-00172)
Supplement: 21-00172-Silverman-Supplement.pdf [file 21-00172-Silverman-Supplement.pdf]

Supplement to: Adams J, MacKenzie MJ, Amegah AK, et al. The conundrum of low COVID-19 mortality burden in Sub-Saharan Africa: myth or reality? *Glob Health Sci Pract*. 2021;9(3). https://doi.org/10.9745/GHSP-D-21-00172

Member States of the African WHO Region[1]

Algeria, Angola, Benin, Botswana, Burkina Faso, Burundi, Cameroon, Cape Verde, Central African Republic, Chad, Comoros, Ivory Coast, Democratic Republic of the Congo, Equatorial Guinea, Eritrea, Ethiopia, Gabon, Gambia, Ghana, Guinea, Guinea-Bissau, Kenya, Lesotho, Liberia, Madagascar, Malawi, Mali, Mauritania, Mauritius, Mozambique, Namibia, Niger, Nigeria, Republic of the Congo, Rwanda, São Tomé and Príncipe, Senegal, Seychelles, Sierra Leone, Somalia, South Africa, Swaziland, Togo, Uganda, Tanzania, Zambia, Zimbabwe.

Member States of the WHO Americas[2]

Antigua and Barbuda, Argentina, Bahamas, Barbados, Belize, Bolivia, Brazil, Canada, Chile, Colombia, Costa Rica, Cuba, Dominica, Dominican Republic, Ecuador, El Salvador, Grenada, Guatemala, Guyana, Haiti, Honduras, Jamaica, Mexico, Nicaragua, Panama, Paraguay, Peru, Saint Kitts and Nevis, Saint Lucia, Saint Vincent and the Grenadines, Suriname, Trinidad and Tobago, United States, Uruguay, Venezuela.

Member States of the South-east Asia WHO Region[3]

Bangladesh, Bhutan, North Korea, India, Indonesia, Maldives, Myanmar, Nepal, Sri Lanka, Thailand, Timor-Leste.

Member States of the European WHO Region[4]

Albania, Andorra, Armenia, Austria, Azerbaijan, Belarus, Belgium, Bosnia and Herzegovina, Bulgaria, Croatia, Cyprus, Czech Republic, Denmark, Estonia, Finland, France, Georgia, Germany, Greece, Hungary, Iceland, Ireland, Israel, Italy, Kazakhstan, Kyrgyzstan, Latvia, Lithuania, Luxembourg, Malta, Monaco, Montenegro, Netherlands, North Macedonia, Norway, Poland, Portugal, Moldova, Romania, Russia, San Marino, Serbia, Slovakia, Slovenia, Spain, Sweden, Switzerland, Tajikistan, Turkey, Turkmenistan, Ukraine, United Kingdom, Uzbekistan.

Member States of the Eastern Mediterranean WHO Region[5]

Afghanistan, Bahrain, Djibouti, Egypt, Iran, Iraq, Jordan, Kuwait, Lebanon, Libya, Morocco, Oman, Pakistan, Palestine, Qatar, Saudi Arabia, Somalia, Sudan, Syria, Tunisia, United Arab Emirates, Yemen.

Member States of the Western Pacific WHO Region[6]

Australia, Brunei, Cambodia, China, Cook Islands, Fiji, Japan, Kiribati, Laos, Malaysia, Marshall Islands, Micronesia, Mongolia, Nauru, New Zealand, Niue, Palau, Papua New Guinea, Philippines, Samoa, Singapore, Solomon Islands, South Korea, Taiwan, Tonga, Tuvalu, Vanuatu, Vietnam.

References:

Supplement to: Adams J, MacKenzie MJ, Amegah AK, et al. The conundrum of low COVID-19 mortality burden in Sub-Saharan Africa: myth or reality? *Glob Health Sci Pract*. 2021;9(3). https://doi.org/10.9745/GHSP-D-21-00172

1. Countries. World Health Organization Regional Office for Africa. Published 2020. Accessed June 21, 2021. https://www.afro.who.int/countries
2. Countries and Centers. World Health Organization; Pan American Health Organization. Published 2020. Accessed June 21, 2021. https://www.paho.org/en/countries-and-centers
3. About WHO in the SEARO. World Health Organization Regional Office for South-East Asia. Published 2020. Accessed June 21, 2021. https://www.who.int/southeastasia/about
4. Countries. World Health Organization Regional Office for Europe. Published 2020. Accessed June 21, 2021. https://www.euro.who.int/en/countries
5. Countries. World Health Organization Regional Office for the Eastern Mediterranean. Published 2020. Accessed June 21, 2021. http://www.emro.who.int/countries.html
6. Where we work. World Health Organization Western Pacific Region. Published 2020. Accessed June 21, 2021. https://www.who.int/westernpacific/about/where-we-work
